# The expanding roles of neuronal nitric oxide synthase (NOS1)

**DOI:** 10.7717/peerj.13651

**Published:** 2022-07-07

**Authors:** Kundan Solanki, Sajjan Rajpoot, Evgeny E. Bezsonov, Alexander N. Orekhov, Rohit Saluja, Anita Wary, Cassondra Axen, Kishore Wary, Mirza S. Baig

**Affiliations:** 1 Department of Biosciences and Biomedical Engineering (BSBE), Indian Institute of Technology Indore (IITI), Simrol, Indore, India; 2 Laboratory of Cellular and Molecular Pathology of Cardiovascular System, Federal State Budgetary Scientific Institution “Petrovsky National Research Centre of Surgery”, Moscow, Russia; 3 Laboratory of Angiopathology, Institute of General Pathology and Pathophysiology, Moscow, Russia; 4 Department of Biology and General Genetics, I.M. Sechenov First Moscow State Medical University (Sechenov University), Moscow, Russia; 5 Department of Biochemistry, All India Institute of Medical Sciences, Bibinagar, Hyderabad, India; 6 Pharmacology and Regenerative Medicine, University of Illinois at Chicago, Chicago, IL, United States

**Keywords:** Nitric oxide synthase, Nitric oxide, Epigenetics, Transcriptional regulation, Vasodilation, Nitric oxide signaling, Cancer, Diabetes, Cardiovascular, Redox

## Abstract

The nitric oxide synthases (NOS; EC 1.14.13.39) use L-arginine as a substrate to produce nitric oxide (NO) as a by-product in the tissue microenvironment. NOS1 represents the predominant NO-producing enzyme highly enriched in the brain and known to mediate multiple functions, ranging from learning and memory development to maintaining synaptic plasticity and neuronal development, Alzheimer’s disease (AD), psychiatric disorders and behavioral deficits. However, accumulating evidence indicate both canonical and non-canonical roles of NOS1-derived NO in several other tissues and chronic diseases. A better understanding of NOS1-derived NO signaling, and identification and characterization of NO-metabolites in non-neuronal tissues could become useful in diagnosis and prognosis of diseases associated with NOS1 expression. Continued investigation on the roles of NOS1, therefore, will synthesize new knowledge and aid in the discovery of small molecules which could be used to titrate the activities of NOS1-derived NO signaling and NO-metabolites. Here, we address the significance of NOS1 and its byproduct NO in modifying pathophysiological events, which could be beneficial in understanding both the disease mechanisms and therapeutics.

## Introduction

**Figure 1:**
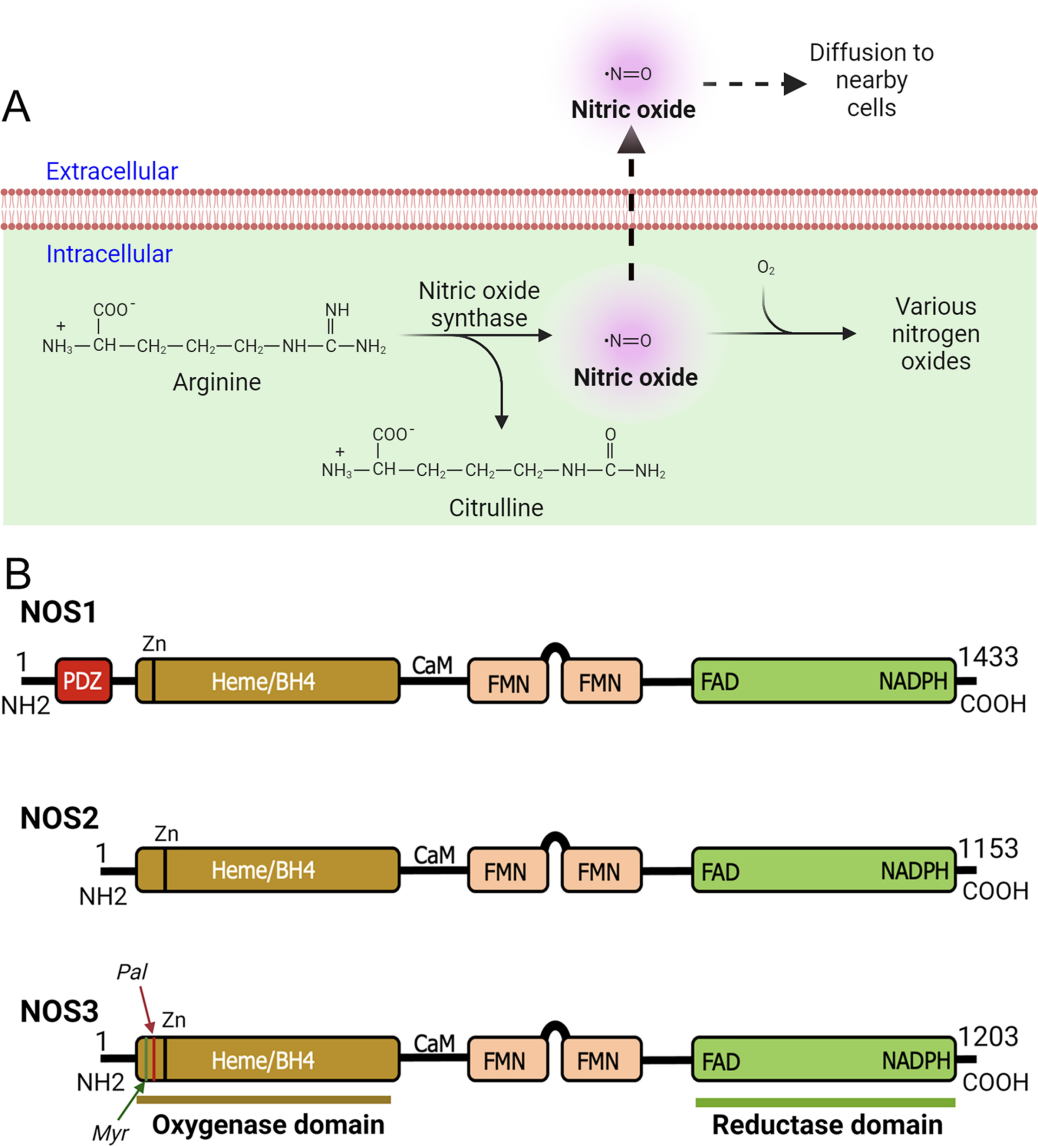
Production of nitric oxide (NO) and functional domains of human NOS1, NOS2, and NOS3. (A) Production of nitric oxide (NO). NOS converts L-arginine to L-citrulline in presence of nicotinamide adenine dinucleotide phosphate (NADPH) and oxygen to produce highly diffusible NO free radical in the tissue microenvironment. (B) Functional domains of human NOS1, NOS2, and NOS3. NOS1 harbors a PDZ domain at the NH2-terminus. The oxygenase and reductase domains are as shown. The oxygenase domain contains heme and tetrahydrobiopterin (BH4) interacting sites, whereas the reductase domain contains interacting sites for FMN, FAD, and NADPH; the FMN domain connects to the oxygenase domain *via* a calmodulin-binding (CaM) domain. The NOS1 and NOS3 proteins contain an autoinhibition segment that interrupts the FMN domain, while NOS2 lacks. Myristoylation (Myr), palmitoylation (Palm), zinc-ligating (Zn) positions are as shown.

The NOS enzymes are primarily responsible for oxidizing L-arginine to L-citrulline in presence of co-factors, secondarily release biologically active NO free radical as a by-product (Fig. 1A) (Morris, 2004). As a highly membrane permeable free radical, NO can modify significant number of molecular targets (Mustafa et al., 2009; Tuteja et al., 2004). Therefore, NO has the ability to regulate diverse biological activities within cells and tissues (Jensen, 2009; Zweier et al., 2010). As NO can modify cysteine residue(s) *via* the redox-based mechanism, therefore, it controls myriad of cellular responses by activating NO-sensitive guanylyl cyclase; transcriptional and translational activities, for example, by interacting with iron-responsive elements; and post-translational modifications, such as ADP ribosylation (Esplugues, 2002; Forstermann & Sessa, 2012; Picón-Pagès, Garcia-Buendia & Muñoz, 2019). Further, NO free radicals (NO*) can collide with superoxide anion (O_2_^−^*) to form peroxynitrite (ONOO^−^) intracellularly (Ischiropoulos, Zhu & Beckman, 1992). Peroxynitrite ONOO^−^ species is equally a highly reactive molecule that can stimulate or modify biological processes including oxidative damage, nitration, peroxidation of lipids, and S-nitrosylation of proteins, lipids, and DNAs, while also generating nitryl and hydroxyl species (Ferdinandy & Schulz, 2003; Ischiropoulos, Zhu & Beckman, 1992). Additionally, ONOO^−^ can induce DNA single-strand breaks thereby activate poly-ADP-ribose polymerase (PARP) (Islam et al., 2015; Szabó, 2006). Superoxide dismutase (SOD) competes with O_2_^−^* in reacting with NO* to form ONOO^−^. Under most physiological conditions NO occurs at a sub-micromolar concentration (Moncada & Higgs, 2006); however, due to the rate constant of ONOO^−^ formation being a few orders of magnitude higher than that of SOD-mediated H_2_O_2_ formation by O_2_^−^, ONOO^−^ is always present even with high expression of SOD, and the formation of ONOO^−^ gradually increase with increasing production of NO* by NOS (Radi, 2018). The compartmentalized action of NO in the tissue microenvironment is diverse and complex. As NO and most of its downstream products are highly reactive and readily diffusible, therefore, it is not surprising to find involvement of NO in many pathophysiological states.

According to their abundance or the tissue type in which they were first discovered, three main NOS enzymes have been described; (a) neuronal NOS (nNOS or NOS1), (b) inducible NOS (iNOS or NOS2), and (c) endothelial NOS (eNOS or NOS3) (Mattila & Thomas, 2014; Moncada, 1999). In addition, there are five different isoforms of NOS1 proteins that are products of alternatively spliced NOS1 mRNAs: NOS1-α, μ, β, γ, and NOS1-2 (Carnicer et al., 2013). Functional domains of human NOS1, NOS2, and NOS3 are as shown (Fig. 1B). With regards to their expression and localization, NOS2 is not constitutively expressed in cells, but its expression can be induced by infection, bacterial lipopolysaccharide (LPS), cytokines, and other agonists (Frost, Nystrom & Lang, 2004; Kim et al., 2005; Zamora, Vodovotz & Billiar, 2000). Although primarily identified in macrophages, the expression of the enzyme can be stimulated in many cell types or tissues, provided that appropriate stimuli are used (MacMicking, Xie & Nathan, 1997; Nathan, 1992). NOS3 is highly enriched in endothelial cells (ECs), responsible for the synthesis of NO to exert vasodilation and to regulate the flow of blood throughout the body. However, this enzyme has also been detected in cardiac myocytes, platelets, certain brain neurons, syncytiotrophoblasts of the human placenta, and kidney tubular epithelial cells (Forstermann et al., 1994; Forstermann & Sessa, 2012). NOS1 is constitutively expressed in specific neurons of the brain (Northington et al., 1996; Tricoire & Vitalis, 2012). Although highly enriched in brain tissues, immunohistochemical analysis showed the occurrence of NOS1 in kidney macula densa (MD), pancreatic islet, vascular smooth muscle cells, spinal cord, adrenal glands, and in peripheral nitrergic nerves (Hall et al., 1994). In mammals, NOS1 is highly enriched in skeletal muscles (Kobayashi et al., 2019; Piknova et al., 2015). As NOS1 is found in particulate and soluble fractions, and localizes to different intracellular compartments, therefore predict its multiple functions in tissue microenvironment (Zhou & Zhu, 2009). However, the underlying molecular mechanisms are crucially unknown.

Discussion on the role and regulation of different isoforms of NOS-derived NO in different cellular contexts have been described elsewhere (Forstermann & Sessa, 2012; Strijdom, Chamane & Lochner, 2009). However, the roles of NOS1 and NO, in non-neuronal tissues have received less attention, to which we have very little insights. We feel that the continued discussion and studies on the roles of NOS1 and NO expressed in non-neuronal pathologies are necessary to synthesize new knowledge. In this narrative review, we identify large gaps in our understanding of the role of NOS1 and NO signaling as they relate to non-neuronal pathologies such as cardiovascular diseases, cancer, obesity, and diabetes. Importantly, we present a reasonable point of view and hypothesis for the general readers in relation to published literature.

## Survey Methodology

For this narrative review, we screened articles from 1991 to 2022 that appeared in PubMed, SCOPUS, and Google. We utilized keywords: nitric oxide synthase, NOS1, NOS2, NOS3, nNOS, iNOS, eNOS, nitric oxide, cardiovascular, obesity, and cancer. To eliminate any potential bias, the searches were conducted by two first authors (KS and SR) independently, and titles and abstracts of the literature were identified by the search criteria. Disagreements were resolved through arbitration with two senior authors (KW and MSB). Cross-references were searched and analysed. Finally, the full texts were read and analysed for relevant information.

No restrictions were used with regards to year or publication type. This narrative review describes and discusses the pathophysiology related to non-neuronal NOS1 and NO activities that were published in the English language. Articles with minimal or no academic significance, editorial comments, individual contributions, and descriptive studies with no quantitative and qualitative conclusions were excluded.

### Regulation of expression of NOS1

The human *NOS1* gene located at the chromosome 12q24.2 (Hall et al., 1994; Newton et al., 2003) is made of 29 exons, 28 introns, and these DNA elements are interspersed over 250 Kb genome. The open reading frame (ORF) of *NOS1* is composed of 4,302 base pairs, and the translation starts at exon-2 and stops at exon-28, thereby give rise to 1,434 amino acid polypeptide species. The *NOS1* gene can produce several alternatively spliced mRNA transcripts (Brenman et al., 1997; Wang, Spitzer & Chamulitrat, 1999). Moreover, literature survey shows complex transcriptional regulation of *NOS1* gene as described below.

The enhancers, promoters and boundary elements (chromatin insulators) spread across the genome can fine-tune the expression of genes. These three major elements can titrate the levels of gene expression precisely, in temporo-spatial manner in response to specific signals. A promoter of gene usually represents a region of a genome that allows for the recruitment of DNA binding transcription factors (TFs) including epigenetic modifiers, where the activities of RNA polymerase facilitate the transcription of a gene. These promoter DNA sequences are located at the 5′ end (upstream) of the transcription initiation site (TSS), but could also be located downstream of TSS. TFs can be positive or negative regulators of gene transcription, however, the promoters, the TSS and the key TFs collaborate, thereby fine tune to regulate the activities of RNA polymerase. Additionally, the promoter DNA sequences can be found in both in forward and in reverse orientations.

**Figure 2:**
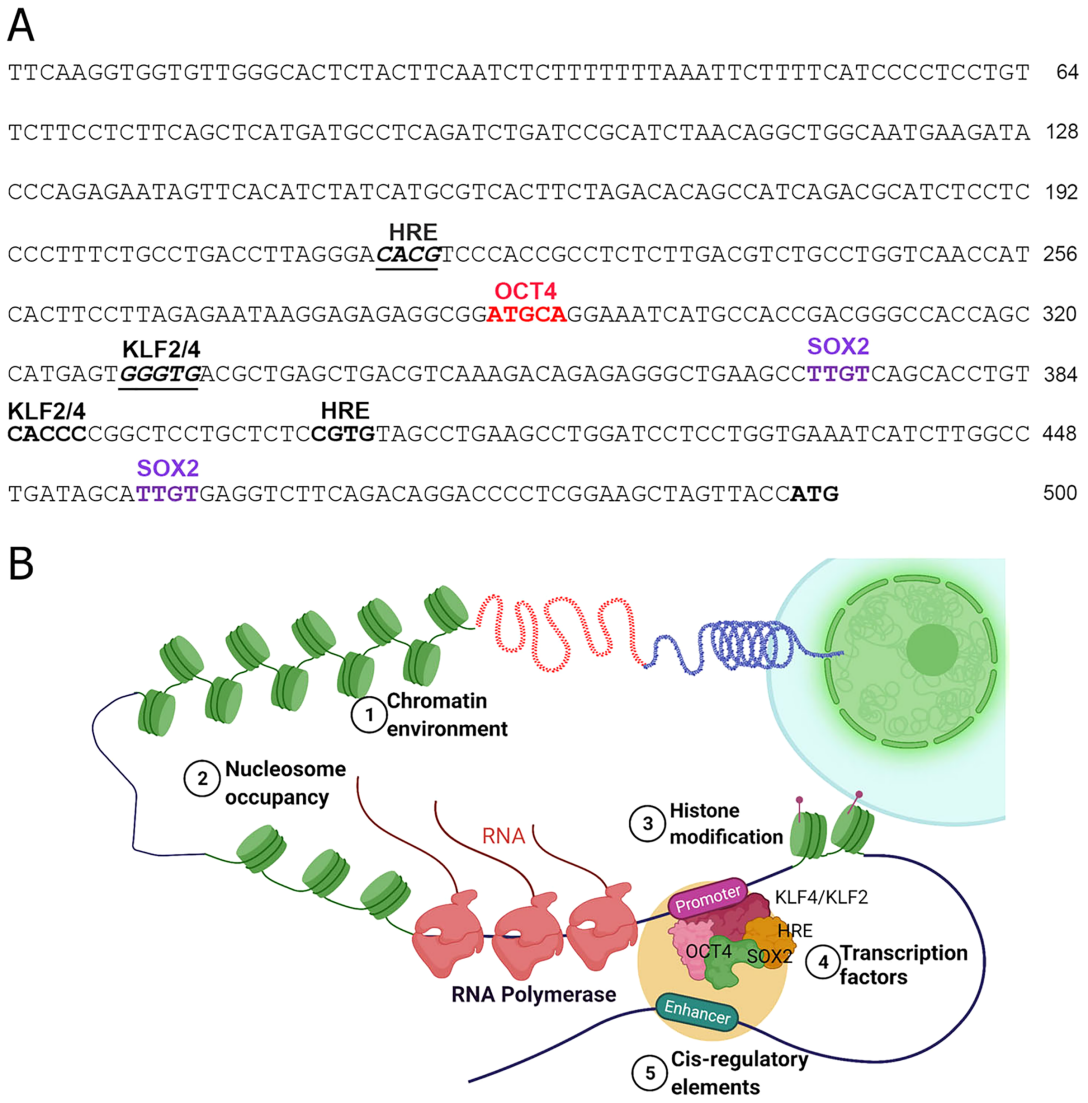
Transcriptional regulation of NOS1. (A) Nucleotide sequence of the human *NOS1*-promoter of -500 bp upstream of the transcription start site (TSS) ATG as shown. The locations of putative binding sites for KLF2/KLF4, Hypoxia Response Element (HRE), OCT4, and SOX2 are indicated. (B) Hypothetical diagram of transcriptional regulation of NOS1 and the potential roles of epigenetic mediators KLF2/KLF4, HRE, OCT4, and SOX2. Epigenetic mediator-induced expression of *NOS1* gene could occur prior to the appearance of genomic instability, thereafter mutation and/or deletion of critical genes could drive tumor cell proliferation, resist apoptosis, together alter NOS1 expression. Additionally, SNPs and polymorphic loci in cis-regulating elements (*e.g*., enhancer) could up-or downregulate NOS1 expression. Expression of NOS1 gene could be measured by RT-PCR, epigenetic modifications by DNA methylation assays or by chromatin immunoprecipitation (ChiP) experiments.

The TFs including the epigenetic modifiers could directly or indirectly regulate the expression of human *NOS1* is not clearly understood. Inspection of 5′ region of the human NOS1-promoter and enhancer suggest that the expression of this gene could be regulated by several TFs, *e.g*., AP-2, TCF/LEF1, CREB/ATF/c-Fos, Ets, p53, and NF-kappa B-like sequences (Hall et al., 1994). In addition, we observed that there are also at least 2 Kruppel Like Factor-2 and -4 (KLF2/KLF4) recognition sites (CACCC) in the human *NOS1*-promoter/enhancer-500 bp upstream of TSS (Fig. 2), and at least 11 binding sites +3.5 kb downstream of exon-1, thereby increasing the complexity of transcriptional regulation of *NOS1* gene by KLF4/KLF2 (Zhang et al., 2005). We also found putative binding sites for OCT4, Hypoxia Response Element (HRE) and Sox2 (Fig. 2). Additional levels of regulation of NOS1 expression are likely due to SNPs and polymorphic loci. Nevertheless, the NOS1-promoter driven green fluorescent protein (GFP) or *Lac-Z* reporter transgenic mice lines should be used with which to study the regulated expression of NOS1 in several pathophysiological setting. The neuronal specific functions of NOS1 are documented very well; however, in the following sections, we highlight the neglected roles of NOS1 in non-neuronal disease settings.

### Roles of NOS1 and NO in cardiovascular disease

The NO is a fundamental modifier of wealth of biological processes in the heart and in the vasculature (Strijdom, Chamane & Lochner, 2009). Owing to its fast reaction kinetics, the roles of NO in different circumstances, at best, is contradictory (Massion et al., 2003; Schulz, Kelm & Heusch, 2004). For instance, in the vasculature, it acts as a vasodilator for smooth muscle cells and an anti-platelet aggregator for platelets (Massion et al., 2003), whereas in cardiomyocytes, it acts as a pro-apoptotic or necrotic if present in excessive amount (Strijdom, Chamane & Lochner, 2009). Thus, the biological effect of NO can oscillate, subject to which NOS polypeptide species is engaged and the bioavailability of NO (Brutsaert, 2003). Specific roles of NOS in cardiovascular systems has been reviewed elsewhere (Strijdom, Chamane & Lochner, 2009).

The effector molecules downstream of NO include ion channels found in the membrane, enzymes, and several key proteins in the mitochondria, cytosol and nuclear compartment (Villanueva & Giulivi, 2010; Zhang, 2017). All three NOS isoforms are abundant in the heart and in atherosclerotic plaques (Pong & Huang, 2015; Wilcox et al., 1997) and have addressed the expression of NOS isoforms in normal and during the progression of atherosclerotic lesions. NOS3 found in quiescent blood vessels is expressed by ECs, where it maintains basal physiological functions. NOS3 expression does not decrease appreciably in early lesions but is significantly decreased in advanced lesions in the EC overlying the atherosclerotic lesion; whereas NOS1 and NOS2 are expressed in early and advanced lesions in macrophages, ECs, and mesenchymal-appearing intimal cells, but not found in a normal quiescent vasculature. The expression of NOS2 was confirmed by immunohistochemical staining, while *in situ* hybridization did not detect its mRNA, suggesting that mRNA level was below the detection limit (Weisz et al., 1994). In mice the loss of NOS2 gene is atheroprotective, conversely, NOS3 provided atheroprotective effect in ApoE^−/−^ (Kuhlencordt et al., 2001; Ponnuswamy et al., 2012). Thus, it can be surmised that NOS2 acts as a pro-atherogenic agent, while NOS3 acts as an atheroprotective agent. Although, there are points and counterpoints regarding the role of NOS1 in atherosclerosis, studies have found that NOS1 acts as an anti-atherogenic agent, which was evident by increased plaque formation and mortality in ApoE/nNOSα double knockout mice (Kuhlencordt et al., 2006). Additionally, NOS1^−/−^ increased the mortality of ApoE^−/−^ mice (Barouch et al., 2003). Reportedly, macrophage NOS1-derived NO mediated the uptake of ox-LDL and foam cell formation with the subsequent expression of adhesion molecules on the endothelial wall, thereby enhancing the inflammatory process (Roy, Saqib & Baig, 2021; Roy et al., 2020). Chakrabarti et al. (2012) studied the effect of NOS1 by tumor necrosis factor (TNF) stimulation in ECs. Accordingly, NOS1-knockdown or inhibition by L-NPA (a selective inhibitor of NOS1) enhanced the inflammatory response by elevating expression of vascular cell adhesion molecule (VCAM)-1, IL-2, and IL-8. NOS1 inhibition also increased the expression of GM-CSF, which plays a major role in the biology of leukocyte production and maturation in bone marrow. NOS1 inhibition did not increase the expression of intercellular cell adhesion molecule-1 (ICAM-1) and anti-inflammatory cytokines IFN-γ and IL-10, suggesting a paradoxical role of NOS1 in ECs. NOS1 inhibition did not alter the mRNA level of VCAM-1, suggesting a posttranscriptional regulatory role of NOS1 in ECs. NOS1-derived H_2_O_2_ had been described as an important vasodilator in ECs, and a reduction in NOS1 expression has been shown to lead to EC-dysfunction in ApoE^−/−^ mice through decrease in H_2_O_2_ level and subsequently decreased vasodilation (Capettini et al., 2011). Ox-LDL reduced NOS1 expression and increased NOS-Ser^852^ phosphorylation, thereby uncoupling of NOS1 with a subsequent decrease in NO and H_2_O_2_ level in ECs, thus generating oxidative stress. Impairment of H_2_O_2_ production in ECs by ox-LDL activated c-Jun and c-Fos, which mediate elaboration of inflammatory cytokines (Navia-Pelaez et al., 2017; Navia-Pelaez et al., 2018). Notably, NOS1 mediated the interaction between ECs and macrophages in the early stage of atherosclerosis. CD40 ligand is expressed on macrophages by ox-LDL activated NOS1. Macrophage secretes soluble factors and increases the expression of the CD40 receptor on ECs. Interaction between CD40-40L generates an inflammatory response and leads to EC-dysfunction (Roy, Saqib & Baig, 2021). A study of myocyte hypertrophy and ventricular stiffness revealed an increased response in NOS1^−/−^ in the progression of both (Barouch et al., 2003), thus signifying the beneficial effect of NOS1 on myocyte hypertrophy. As the (a) expression of NOS1 was found in early and advanced atherosclerotic plaques (Wilcox et al., 1997) and (b) the concentration of NO determines the pro-or anti-inflammatory processes (Brutsaert, 2003), studies explaining the role of NOS1-derived NO in early and advanced atherosclerotic plaque by modulating the concentration of NO and its subsequent effect on atherosclerosis plaque could help clarify the role of NOS1-derived NO in these processes. However, laboratory animal experiments have their own limitations as these experiments are often carried out in inbred strains of mice coupled with restrictive dietary regiments.

NOS1 is localized on the membrane vesicles of the smooth endoplasmic reticulum (SER) in cardiac muscles (Xu et al., 1999) that modulate contractility of cardiac muscle and regulation of intracellular Ca^2+^ movement (Carnicer et al., 2013). NOS1-derived NO inhibited Ca^2+^ influx into cardiomyocyte *via* regulation on L-type Ca^2+^ channel (LTCC) present on the cell membrane. It increased Ca^2+^ reabsorption in the SER by increasing phospholamblan phosphorylation and regulating the release of Ca^2+^ from the SER through S-nitrosylation of the ryanodine receptor (RyR) Ca^2+^ release channel present on the SER (Carnicer et al., 2013). *NOS1* gene deletion decreased the S-nitrosylation of RyR (Wang et al., 2010). However, these contrasting results suggest the ability of NOS1 in regulating the excitation-contraction (EC) coupling response in cardiomyocytes. Enhanced contraction and relaxation in left-ventricular cardiomyocytes in NOS1^−/−^ mice compared to wild-type mice have been documented (Ashley et al., 2002), which was subsequently confirmed by others (Burger et al., 2009; Dawson et al., 2005; Sears et al., 2003), although some studies demonstrated otherwise (Barouch et al., 2003; Wang et al., 2010). This signifies presence of other variable factors, which could modify the NOS1-derived physiological responses, *e.g*., temperature modulated NOS1 behavior on EC coupling (Dulce et al., 2015). Temperature has been known to modulate NO production by affecting NOS activity (Venturini et al., 1999). Impairment in Ca^2+^ handling, such as Ca^2+^ leak, contribute to impairment in contractibility by depleting Ca^2+^ storage in the SER. Xanthine oxidase (XOR) and NOS1 have been shown to co-localize on the SER in proximity to RyR2. NOS1-derived NO mediates the inhibitory effect on XOR and thus regulates RyR2, and a decrease in NOS1 leads to a rise in the generation of superoxide anion (Khan et al., 2004) and the dysregulation of RyR2-mediated Ca^2+^ release. In wild-type cardiomyocytes, a reduction in temperature increased Ca^2+^ leak due to increased ROS generation through XOR activity and NOS1 uncoupling, therefore decreasing the S-nitrosylation of RyR2 and affecting the contractibility of myocardium. Every time NO collides with superoxide anion, this event generates peroxynitrite, which induces more damage to RyR2 compared to ROS (Khan et al., 2004; Kohr et al., 2012). Mice that lack denitrosylation machinery (GSNOR^−/−^) showed reversed cooling-induced ROS generation and calcium leak. In a NOS1^−/−^ mice model, higher temperature (>30 °C) increased Ca^2+^ leak and elevated ROS (Dulce et al., 2015). Therefore, this observation indicated that the effect of NOS1 on cellular activity cannot be studied by modulating the intrinsic parameters only, as extrinsic factors could also be a major player.

Further complicating the picture, epigenetic modification in the NOS gene and in the promoter/enhancer regions could also modulate disease states, because such modification can alter gene expression, generation and bioavailability of NO in the cells and tissues (Das, Ravikanth & Sujatha, 2010). Methylation of the NOS1 gene plays an essential role in atherogenesis in children (Breton et al., 2014). The methylation pattern of 16 CpG loci located within NOS1, NOS2A, NOS3, ARG1, and ARG2 genes was analysed in 377 children, with a history of carotid intima-media thickness (CIMT), and linear regression was plotted with CIMT measurement. CIMT was found to increase by 1.2 μm for every 1% increase in the average DNA methylation of the NOS1 gene (*p* = 0.02) (Breton et al., 2014). Methylation patterns on the non-CpG island and their correlation to CIMT were addressed as well, in which the subject with high mean methylation on the non-CpG island of the NOS1 gene had a 15.8 μm higher measurement of CIMT (*p* = 0.004). Although extrapolation of this study’s findings on larger cohorts would clarify the correlation of CIMT with NOS1 methylation status, the findings on these subjects signify the importance of epigenetic modification on NOS1 gene. In addition, the occurrence of single nucleotide polymorphism (SNP) in NOS1 genome has been suggested to elevate the risk of cardiovascular disease, for example, in coronary heart disease and in hypertension (Fiatal & Adany, 2017). In a total of 3,351 individuals (560 cases of coronary heart disease (CHD) and 2,791 controls in which 1,158 individuals were hypertensive) genotyped for 58 SNPs in NOS genes, the presence of NOS1 SNP rs3782218 showed a positive correlation with disease pathogenesis in both cases of CHD and hypertension, whereas another NOS1 SNP (rs2682826) showed a positive correlation with CHD but not with hypertension (Levinsson et al., 2014). Thus, early detection of these SNPs in individuals can serve as an effective marker for the pathogenesis of CHD and hypertension, leading to early treatment. Sudden cardiac death caused by abrupt decline of heart function within 1 h of the onset of these symptoms (Sara et al., 2014). In this regard, coronary artery disease is likely the main driver of sudden cardiac death, and most deaths harbor genetic variations (Chang et al., 2013). For example, SNP in NOS1 adaptor protein (NOS1AP; also known as CAPON) was linked with QT prolongation phenomena (delayed ventricular repolarization) in the general population (Kao et al., 2009) and increased sudden death in patients with type 1 QT (Tomás et al., 2010). NOS1AP is expressed in the cardiac myocyte, which interacts with NOS1 to suppress the sarcolemma L-type calcium channel (LTCC) *via* the S-nitrosylation, thereby enhance Iκr current, accelerating cardiac repolarization (Treuer & Gonzalez, 2014). Analysis of SNP in NOS1AP could serve as an indicative marker for the estimation of potential sudden cardiac death and for early treatment in patients. Nevertheless, genetic experiments with mouse model mimicking human SNPs with the use of CRISPR/Cas9 technology, thereby introducing precise SNP variations analogous to human counterparts, might be useful in determining the precise roles of SNPs in the above-described cardiovascular diseases. SNPs that are found within the promoter/enhancer regions have been hypothesized to increase or decrease or even create a new binding site for TFs, thereby controlling RNA-polymerase activities to turn on the transcription of downstream target genes. Careful genetic and biochemical studies are needed to address such possibilities.

### What is known about the roles of NOS1 and NO expression in Cancer?

The roles of NO in cancer, at best, represent crucially important unknowns. For example, low levels (<100 nM) of NO are thought to enhance tumor progression and metastasis, while high levels (>300 nM) of NO induce cell cycle arrest, senescence, and apoptosis (Choudhari et al., 2013). All three NOS have the potential to either inhibit or promote cancer growth by adjusting the level of NO. Accumulation of NOS1-derived NO in various types of cancer cells seemingly plays a crucial role in tumor progression, however, mechanistic details are far from understood (Hamaoka et al., 1999). NO can modify the DNA damage and repair mechanisms in tumor by upregulating p53, poly(ADP-ribose) polymerase (PARP), and DNA-dependent protein kinase (DNA-PK), thereby modulating the cellular apoptosis (Weiming et al., 2002). In the following sections, we describe the cellular mechanisms governed by NOS1 in the progression of a subset of tumors.

#### Cervical cancer

Cervical cancer is the fourth most common form of cancer that occur in women (Vaccarella et al., 2014). In these patients, the expression of adenosine triphosphate (ATP)-binding cassette sub-family G member 2 (ABCG2) correlates strongly with anti-neoplastic drug resistance and migratory/invasive cancer cell phenotypes (Robey et al., 2007). Accordingly, the mRNA expression of NOS1 and ABCG2 were analyzed in 40 human cervical cancer tissues and 20 control tissues (Ding et al., 2019). The expression levels of NOS1 and ABCG2 mRNAs increased compared to the normal control group with a mean difference of 2.63 and 2.02 times, respectively (*p* < 0.05), and therefore strongly correlate (r-value, 1.246; *p* = 0.014). In an experiment, NOS1-depletion decreased ABCG2 protein level, in contrast, ABCG2-depletion did not change the NOS1 protein level, indicating that NOS1 is upstream of ABCG2 in cervical cancer cells. In their study, NOS1-depletion significantly decreased the proliferation and increased the apoptosis of cervical cancer cells (*p* < 0.05), suggesting pro-life roles of NOS1 in cervical cancer (Ding et al., 2019). In a separate study, a positive correlation was reported between ABCG2 and NOS1 expression in cisplatin-induced chemoresistance in ovarian cancer (Li et al., 2019). Thus, delineating the signaling cascade involved in the regulation of ABCG2 by NOS1 would unravel clearer mechanism into the specific role of NOS1 in cervical cancer cells, which could be an effective target in the management of cervical cancer.

#### Melanoma

Perturbation of immune system is a hallmark of neoplastic transformation. For example, type I interferon (IFN) dysfunction allows for immune escape and tumor metastasis. In contrast, constitutive activation of IFN signaling affords increased immune surveillance against cancer (Budhwani, Mazzieri & Dolcetti, 2018). STAT1 phosphorylation by IFNα activates the intrinsic signaling cascade, which activates the expression of interferon-stimulated genes (ISGs) (Critchley-Thorne et al., 2007). In melanoma as well as colon and breast carcinoma, exposure to peripheral blood mononuclear cells (PBMC) with IFN-α *ex vivo*, activated ISGs, reduced phosphorylation of STAT-1 and decreased immune response, which was due to decreased expression of ISGs (Critchley-Thorne et al., 2007, 2009). Studies identifying the link between the genetics of cancer patients and its effect on IFN signaling in PBMC can help in predicting the clinical outcome in the patient due to modulation in immunosurveillance. Analysis of the transcription level of the genes in different melanoma patients dictated that a decrease in IFN signaling in PBMC correlates with amplification of NOS1 locus and its expression in melanoma cells (Liu et al., 2014). The same research group further addressed the mechanism of action of NOS1 in the suppression of IFN signaling in melanoma cells (Xu et al., 2019). Histone deacetylase 2 (HDAC2) plays a key role in the expression of ISGs at the transcriptional level, as HDAC2-knockdown decreases the response to IFNα (Icardi, De Bosscher & Tavernier, 2012). Accordingly, NOS1 mediated S-nitrosylation of HDAC2 at Cys-262 and -274 (Nott et al., 2008). In a controlled experiment, HDAC2 deacetylated H4K16, thereby recruiting RNA polymerase II to the promoter and lead to expression of ISGs in melanoma cells. In this study, NOS1 decreased the IFNα response by S-nitrosylating HDAC2 at position Cys^262^/Cys^274^ (Nott et al., 2008). S-nitrosylation of HDAC2 by NOS1 decreased the binding of HDAC2 to STAT1, thereby reduced HDAC2 recruitment to the promoter of ISGs, subsequently decreased deacetylation of H4K16. A mutant form of HDAC2 (Cys^262^/Cys^274^) that cannot be nitrosylated, reversed NOS1-mediated ISG inhibition, reduced NOS1-induced lung metastasis, and inhibited tumor-homing lymphocytes in a mouse models of melanoma (Xu et al., 2019). Thus, NOS1 mediated repression of ISGs through HDAC S-nitrosylation serves as a novel pathway in the progression of melanoma, and studies identifying the targets of NOS1 would help in understanding the cellular mechanism and in designing better immunotherapeutic strategies to target melanoma.

#### Non-small cell lung carcinoma (NSCLC)

The lung tumors such as NSCLC, lung adenocarcinoma, and lung squamous cell carcinoma, together add up to 80% of the lung cancer incidences (Goldstraw et al., 2011). A hallmark of tumor microenvironment is the accumulation of stromal fibroblasts, by interacting with tumor cells, these cells can accelerate tumor growth and metastasis (Ji et al., 2018; Quail & Joyce, 2013). Chemo-attractant cytokines, called the chemokines control cellular behavior such as cell migration during organogenesis and immune surveillance (Song et al., 2010). CXCL14 (C-X-C Motif Chemokine Ligand 14) is expressed in various types of cancer and in stromal cells (Kleer et al., 2008; Schwarze et al., 2005; Wente et al., 2008; Zeng et al., 2013). The specific role of CXCL14 is likely context dependent, in that it can promote or regress depending upon which cell type expresses the ligand. For instance, when expressed by the stromal fibroblasts, it showed a tumor-promoting effect (Augsten et al., 2009), in contrast, it acted as an antitumor when expressed by carcinoma cells (Gu et al., 2012). Immunohistochemical combined with morphometric analyses were conducted to address the relevance of expression and correlation of CXCL14 and NOS1 in Stage I–IIIA NSCLC patients. In their analyses, CXCL14 was expressed by stromal fibroblasts as well as in cancer cells, while NOS1 was seen only in cancer cells, but not in stromal fibroblast. However, NOS1 level in cancer cells was strongly associated with CXCL14 level in stromal fibroblasts, showing a decrease in progression-free survival (PFS) and overall survival (OS) (Ji et al., 2018). While CXCL14-expressing fibroblasts promoted the growth of prostate tumors by NOS1 secretion; in NCSLC, the expression of CXCL14 and NOS1 served as surrogate markers of cancer progression (Augsten et al., 2014). Thus, more research is warranted to understand how CXCL14 in stromal fibroblasts modulates the level of NOS1 expression in relation to NSCLC disease states. In addition, it would be a rewarding endeavor to carry-out loss- and gain-of-function experiments, thereby titrating down- or up- the levels of CXCL14 to address how CXCL14 levels in stromal cells alters the fate of NSCLC. Indeed, if CXCL14 is required for tumor growth and metastasis, CXCL14 neutralizing antibody could have therapeutic use in the treatment of NSCLC.

#### Colon cancer

The N-terminus of the NOS1 protein harbors a PDZ (PSD-95/Dlg/ZO-1) domain, which mediates its subcellular localization (Zhou & Zhu, 2009). In their study, NOS1 protein translocated to mitochondria through Hsp90, as geldanamycin (C-terminal inhibitor of Hsp90) treatment inhibited mitochondrial localization. The mitochondria produced reactive oxygen species (ROS) in response to cisplatin, which mediated apoptotic pathway through the release of Cytochrome-c. Additionally, overexpression of mitochondrial NOS1 (mNOS1) inhibited the formation of superoxide anions and the expression of Cytochrome-c after cisplatin treatment. Thus, mNOS inhibits cisplatin-induced apoptosis *via* mitochondrial ROS suppression (Wang et al., 2019). Superoxide dismutase 2 (SOD2) helps in the removal of mitochondrial superoxide (Wang et al., 2018). Anticancer drugs increase ROS production to a toxic level in cells, thus mediating tumor cell death (Aggarwal et al., 2019). Sirtuin 3 (SIRT3) maintains mitochondrial ROS below toxic level, thereby optimizing tumor cell survival and proliferative mechanism. Enhanced SIRT3 activity enhanced cancer cell resistance to radiation, as well as by chemotherapeutic drugs (Liu et al., 2015). mNOS1 increased SIRT3 activity, thereby attenuating mitochondrial superoxide- and cisplatin-induced apoptosis (Wang et al., 2019). Thus, Hsp90 induced translocation of mNOS1 to the mitochondria, accordingly enhanced SIRT3 activity to decrease cisplatin-induced ROS generation, suppressed intrinsic apoptosis pathway and promoted tumor growth (Wang et al., 2019). Thus, NOS1 limits the magnitude of toxicity of superoxide in mitochondria, thereby providing survival advantage. However, sophisticated cellular and molecular experiments are needed to determine the roles of NOS1 and NO signaling in colon cancer progression.

### What is the role of NOS1 and NO signaling in diabetes?

Diabetes mellitus (DM) is a metabolic syndrome defined by chronic hyperglycemia occurring owing to defects in insulin secretion or action mechanisms, or a combination of both (American Diabetes, 2015). The magnitude and kinetics of hyperglycemia can give rise to EC dysfunction and blood vessel complications. These complications are, (a) microvascular: diabetic kidney disease, retinopathy, and neuropathy; and (b) macrovascular: coronary artery disease, peripheral vascular disease, and stroke, that are linked with morbidity and mortality events in diabetic patients (Assmann et al., 2016). One of the underlying factors in vascular pathophysiology is NO, where hyperglycemia has a major impact (Heltianu & Guja, 2011; Vareniuk et al., 2009; Yamagishi & Matsui, 2011). Additionally, the incidences of DM have been linked with alterations in NO-mediated vasomotor dysfunction (Komers et al., 2000a; Pieper, 1998). NO function has been shown to regulate systemic and local hemodynamics (Dellamea et al., 2014; Khamaisi et al., 2006; Komers et al., 2000a). NOS1-derived NO in MD cells and its role in the regulation of glomerular hemodynamic and renal dysfunctions are of great interest to nephrologists; accordingly, several studies reported NOS1-mediated NO in controlling the DM disease states (Khamaisi et al., 2006; Komers et al., 2000a; Komers et al., 2000b; Komers et al., 2004; Zhang et al., 2019). Diabetes nephropathy (DN) represents one of the main chronic complications associated with T1DM and T2DMs, together represent the leading causes of renal failure, defined by an increased glomerular filtration rate (GFR), perfusion, and renal hypertrophy (Khamaisi et al., 2006; Zhang et al., 2019). Although the role of NOS1-derived NO is not fully elucidated as it relates to pathogenesis of glomerular hyperfiltration in diabetes, a few studies have delineated its significance and the underlying biological mechanism. A recent study reported a SGLT1 (Sodium-Glucose Cotransporter 1)-NOS1-TGF signaling axis mediating acute hyperglycemia-associated glomerular hyperfiltration, where NOS1 and SGLT1 may prove to be key therapeutic targets (Zhang et al., 2019). This mechanism suggests that acute hyperglycemia-induced hyperfiltration increases luminal glucose at MD, leading to an increase in the expression and activity of NOS1 *via* SGTL1, thus blunting the tubuloglomerular feedback (TGF) response and promoting glomerular hyperfiltration. The authors observed that in both mice and human renal cortices, high glucose upregulated NOS1 level and increased phosphorylation of NOS1 at Ser^1417^. Interestingly, MD-*NOS1* knockout showed no defect in NO generation and effect upon glucose addition to the MD perfusate, as well as no significant TGF response after glucose addition to tubular perfusate. Eventually, acute hyperglycemia-induced elevation in GFR was significantly attenuated, which suggests the key role of NOS1 in mediating glucose-induced hyperfiltration (Zhang et al., 2019). As discussed above the NOS1-mediated NO production is a major factor in the pathogenesis of renal hemodynamic changes in the early course of diabetes and considered it as the major denominator in the NO mechanisms in this pathology (Komers et al., 2000a, 2004, 2000b). In their previous study, the inhibition of NOS1 by the administration of *S*-methyl-l-thiocitrulline (SMTC) both in control and experimentally induced diabetic rats increased blood pressure, but not in normal rats; additionally, diabetic rats showed elevated renal hemodynamic response, suggesting diabetic rats are more sensitive to this inhibition (Komers et al., 2000a). Subsequently, these authors reported that STZ-induced diabetic rats have increased the number of NOS1-positive cells; and in these rats SMTC administration normalized the elevated GFR in diabetic rats but had no effect on non-diabetic rats, defining the NOS1-specific role in renal hemodynamics in diabetes (Komers et al., 2000b). Additionally, the same group of authors published a separate study to analyze the nephroprotective role of NOS1 and addressed the progression of renal injury in terms of proteinuria and glomerular sclerosis in uninephrectomized diabetic and non-diabetic rats (Komers et al., 2004). This time, the long-term impact of the NOS1-selective inhibitor SMTC was assessed, revealing a modest effect where the renal injury was delayed in diabetic mice, whereas no beneficial effects were observed in normal rats; rather, it increased glomerulosclerosis. Further, SMTC-treated diabetic rats displayed a reduction in weight gain (Komers et al., 2004). However, cyclooxygenase-2 (COX-2) expression and activity increase in renal injury, and its action has been implicated in the pathophysiology of such conditions, while NOS1 derived NO in MD is reported as one of the COX-2 activators (Cheng et al., 2000; Wang et al., 2000). Thus, NOS1 inhibition can modulate COX-2 activity to provide therapeutic benefits in conditions such as proteinuria and glomerulosclerosis. However, SMTC inhibition of NOS1 had no effect in COX-2 level in the renal cortex, and this finding does not represent the COX-2 role in mediating the beneficial effect *via* NOS1 inhibition in diabetic rats; nevertheless, the effect of the NOS1-COX-2 axis during nephropathy cannot be excluded (Komers et al., 2004).

Excluding DN, the role of NOS1 has also been studied in diabetic cardiomyopathy (DCM). DCM is characterized by cardiac muscle dysfunction and change in cardiomyocyte architecture, caused mostly due to altered glucose metabolism homeostasis, characterized by cardiac muscle contractility dysfunction (González, Treuer & Novoa, 2016; Pappachan et al., 2013). As diabetes progresses, systolic dysfunction develops, the cardiac muscle deteriorates, and the production of oxidative stress increases. An increase in oxygen species is directly proportional to NOS uncoupling, which can be reversed for NOS1, upon treatment with cofactor tetrahydrobiopterin (BH4) and sepiapterin, thereby reconfiguring the production of superoxide rather than NO (Ansley & Wang, 2013; González, Treuer & Novoa, 2016). T1DM predicts adverse cardiovascular events, *e.g*., the incidence of heart failure is 2–3 fold higher in diabetic than in normal cohorts (Rosano, Vitale & Seferovic, 2017). Reduction of myocyte and cardiac contractility is the hallmark of DCM (Amour et al., 2007). Due to NOS1 activity in the regulation of RyR2 S-nitrosylation (Wang et al., 2010), β-adrenergic in the heart, and preventing cardiac dysfunction (Ashley et al., 2002), and the roles of NOS1-derived NO in DCM has been reported. Increased NO concentration could develop EC-dysfunction and atherosclerotic complications in DM; however, it may precipitate into insulin resistance. In one study, *NOS1* knockout showed insulin resistance, hypertension, and dyslipidemia (Duplain et al., 2001). The subsequent hyperglycemia, hyperlipidemia, and insulin resistance, together, augmented oxidative stress in the diabetic cardiac muscles. In diabetic vascular and cardiomyopathy, the oxidation of NOS cofactor BH4 and dysfunctional activity of NOS are known. In a recent study, an increase in cardiac muscle BH4 prevented and reversed left ventricular remodeling and systolic dysfunction associated with diabetes, *via* a mechanism whereby NOS1-derived NO mediated an increase in insulin-independent myocardial glucose absorption and consumption (Carnicer et al., 2021).

Independent studies have reported that in DCM, NOS1-derived NO mediates the effect of the β3-adrenoceptor (β3-AR) being involved in an altered positive ionotropic activity to β-adrenoceptor agonist stimulation (Birenbaum et al., 2008; Moens et al., 2010; Niu et al., 2012). NOS1 also exerts a crucial role in the mechanism of this disease by the β3-AR-NOS1-RyR2 specific pathway. Inhibition of NOS1 leads to normalization of adverse conditions. In cardiomyocytes, NOS1 is coupled to β3-AR/caveolin3 (Cav3) complex present in the sarcolemma. NOS1 translocation from the sarcoplasmic reticulum (SR) to Sarcolemma caveolae decreased the nitrosylation of RyR2 and activated to release uncontrolled Ca^2+^ induced arrhythmias (Gonzalez et al., 2007). Regular exercise plus insulin treatment has been regarded as an effective therapy for avoiding the complications of type 1 diabetes (T1DM) (Gulve, 2008). Combined insulin treatment with exercise training was studied on diabetic male Wistar rats for 8 weeks to deduce its effect on baseline heart physiology and NOS1, β3-AR, and RyR2 signaling pathways. Experiments were conducted in four diabetic rodent cohorts: (1) with no treatment, (2) with insulin treatment, (3) trained with exercise, and (4) trained with exercise that received insulin. The diabetic control group showed decreased basal systolic and diastolic cardiac function, and RyR2 expression, with increased β3-AR and NOS1 expression. However, combined treatment in Group 4 did not display normalized diastolic pressure but showed normalized systolic pressure and induced increased RyR2 expression, and this effect was higher than in Group-2 and -3 rodents. Complete normalization of β3-AR and downregulation of NOS1 were observed in all three treatment cohorts (Lahaye et al., 2011).

Polymorphism in NOS1 in DM and the genetic variant of NOS1 have been reported. For example, common variation in *NOS1AP* loci is associated with T2DM in African American and Caucasian cohorts; specifically, the study found that variation in *NOS1AP* rs12742393 was strongly linked with susceptibility to T2DM (Wang et al., 2014). Analyses of 79 SNPs in a group of Shanghai Chinese subjects comprised of 1,691 diabetic and 1,720 normal individuals, an association between NOS1AP SNP rs12742393 and T2DM were observed. Although this variant locus may not be a clinically relevant to T2DM incidence, its effect cannot be neglected (Hu et al., 2010). Nevertheless, the roles of SNPs are far from clear, therefore, novel techniques are needed to address the roles of SNPs, not only in cardiovascular disease and diabetes, but also in many neglected diseases as well.

### What is the role of NOS1 in obesity?

Obesity is a major health concern worldwide (Yazdi, Clee & Meyre, 2015) as risks for hypertension, diabetes, metabolic syndrome, and stroke are increased in these patients (Ma et al., 2012). Sedentary lifestyle and genetic variations are considered main drivers of obesity (Yazdi, Clee & Meyre, 2015). In this regard, obesity is associated with an imbalance of energy, where the consumption of calories is higher than those required by bodily processes (Sansbury & Hill, 2014; Tseng, Cypess & Kahn, 2010). The homeostatic steady state between energy consumption and expenditure is complicated, likely modulated by several factors, however, understanding underlying mechanism of this metabolic disorder and its key players is crucial. To this end, NO is likely to be a proximal cause of obesity (Sansbury & Hill, 2014), as obese rodents and human cells reportedly have elevated levels of BH2, an oxidized form of BH4. BH4 deficiency is a major factor in the EC damage and dysfunction that occur during obesity, which uncouples the ability of NOS1 to superoxide generation (Ding & Triggle, 2010; Sánchez et al., 2012; Sansbury & Hill, 2014). The underlying facts of NOS1-derived NO in obesity are described as drivers of hyperphagia, a phenomenon in which individuals desire to increase food intake (Sansbury & Hill, 2014). Mice cohort receiving a high-fat diet had an increase in NOS1 in their aortas, and the induction of NOS1 was demonstrated to be due to leptin stimulation (Benkhoff et al., 2012; Sansbury & Hill, 2014). Interestingly, a study stated that leptin deficiency decreases the expression of NOS1 and NO, with an increase in xanthine oxidoreductase (XOR) activity and oxidative stress, thus mediating imbalance in nitroso-redox generation and generating myocardium dysfunction in obesity. Mice lacking leptin (*ob/ob*) develop cardiac hypertrophy, increased apoptosis of cardiac muscle cells, and decreased survival. Nitrate and nitrite production were reduced in myocardium of *ob/ob* mice. Leptin treatment restored NOS1 protein in *ob/ob* mice. The ratio of GSH/GSSG was decreased, suggesting an increase in oxidative stress in *ob/ob* mice (Saraiva et al., 2007). In another study, the production NOS1 increased, but decreased catalytic activity of NOS1 in pancreatic beta-cell hyperactivity, in insulin-resistant rats and islets of obese human individuals. Islets from Zucker *fa/fa* rats showed increased sensitivity to glucose, with subsequent utilization and oxidation. These results in the hypersecretion of insulin due to the fatty acid esterification resulting from increased glucose responsiveness in beta cells (Mezghenna et al., 2011). Therefore, these studies suggested that 50–70% of the variation in body weight can be attributed to genetic variation. In a Korean population, three SNPs (rs2293048, rs9658490, and rs4766843) were described in the NOS1 gene; two of which (rs2293048 and rs9658490) showed close association with obesity. A total of six haplotypes among the SNPs of NOS1 (CCG, CGA, TCA, TCG, TGG, and CCG, and TCG and TGG) were associated with increased susceptibility to obesity (Park et al., 2016). Therefore, NOS1 in the regulation of obesity warrants more in-depth studies to reveal the significance of NOS1-derived NO in relation to obese subjects.

### What is NOS1 doing in other diseases?

In addition to the above-mentioned diseases, the role of NOS1-derived NO has also been described in other pathologies, such as sepsis, achalasia, and infantile hypertrophic pyloric stenosis. NO modulates leukocytes activities and the response of the immune system to infection, sepsis, or septic shock (Baig et al., 2015; Duma et al., 2011). NOS1 also occurs in the epithelium and microvasculature of the gastrointestinal tract (Takahashi, 2003), in the myocytes of skeletal muscle (Baldelli et al., 2014), in the bronchial epithelium (Shan et al., 2007), in mast cells and neutrophils (Yoshimura et al., 2012). During sterile peritonitis, NOS1^−^/^−^ mice displayed increased rolling of leukocytes and adhesion to the post-capillary venule endothelium and its subsequent migration to the peritoneal cavity. Although NOS1^−^/^−^ mice displayed increased migration of leukocytes in chemical peritonitis and live bacterial peritonitis, a genetic deficiency in NOS1 lead to increased mortality (Cui et al., 2007), indicating its pro-life function.

In a mouse model sepsis, increased elaboration of proinflammatory cytokines TNF-α, IL-1β, IL-12, and IL-17 were reported (Schulte, Bernhagen & Bucala, 2013). NO plays a pivotal role during sepsis, exemplified by hypotension (low blood pressure) or hypo-responsiveness (decrease in the response to vasoconstrictors) (Levy et al., 2012). NOS1 and soluble guanylate cyclase are expressed at high levels in vascular tissue during sepsis, and the blockade of NOS1 inhibits cGMP production, indicating a physical interaction between the two. Pharmacological blockade of NOS1 by 7-nitroindazole (7-NI) or S-methyl-L-thiocitrulline (SMTC) decreased hypo-responsiveness and increased vasoconstriction in a mouse model. Thus, inhibiting NOS1 may help to increase the effect of vasopressors during the late sepsis (Nardi et al., 2014). In contrast, NOS1-derived NO regulated downstream signaling and cytokine expression in macrophages (Baig et al., 2015). This investigation suggested that NOS1-derived NO plays an early role and targets the SOCS1 protein, leading to its degradation. SOCS1 is mainly responsible for the proteasomal degradation of the TIRAP protein, which eventually disrupts TLR-mediated inflammatory signaling and inactivate TF NF-κB. However, the inhibition of SOCS1 by NOS1-derived NO activated NF-κB mediated elaboration of pro-inflammatory cytokines (Baig et al., 2015; Rajpoot et al., 2021). Therefore, it is conceivable that the complete ablation of NOS1 could alter signaling pathways at several cellular and molecular levels in the tissue microenvironment.

Achalasia is a rare disorder, wherein a patient experience trouble passing food or liquid from the esophagus into the stomach. Reduced peristaltic action of the esophagus emerges as smooth muscles fail to relax, thereby causing the lower esophageal sphincter to remain closed, inhibiting the passage of food (Pandolfino & Gawron, 2015). NO activity can delay the contraction of the distal esophagus and relaxing the lower esophageal sphincter to allow peristaltic action (Ghoshal, Daschakraborty & Singh, 2012). A study of the genetic polymorphism of NOS correlated with the presence of nNOS29 C/T with achalasia (Singh et al., 2015). NOS1 C/T allelic variant in exon 29 that resides in the untranslated region of the gene likely alters the NOS1 mRNA stability (Levecque et al., 2003). Thus, the loss of NOS1 predicts poor prognosis of achalasia. Analysis of two siblings with infant-onset achalasia displayed bilateral premature stop codon in the NOS1 gene, which resulted in defects in folding, cofactor binding, and NO production, suggesting the importance of NOS1-derived NO in the prevention of achalasia (Shteyer et al., 2015). Nevertheless, additional studies in aged populations should provide a clearer insight into its role in achalasia.

Infantile hypertrophic pyloric stenosis (IHPS) results from hypertrophy (increase in cell size) of the pyloric muscle and is the most common disease of the gastrointestinal tract in infants (Galea & Said, 2018). NOS1 is the main source of NO in the gut (Saur et al., 2000) and plays a major role as a neurotransmitter in the gastrointestinal tract, causing relaxation of smooth muscle of the tract (Takahashi, 2003). Genetic disequilibrium of the NOS1 variant locus has been found to pose a susceptibility to IHPS (Chung et al., 1996). Genetic analysis of the NOS1 gene conducted in IHPS patients revealed two different mutations, (a) one between the 21^st^ and 22^nd^ exomes, which affected splicing, and (b) another at the 3′ untranslated region, which affects the binding of TFs. The first variant decreased the mRNA expression of NOS1 in IHPS patients (Jabłoński et al., 2016). However, there are contrasting results for NOS1 polymorphisms in IHPS patients. Genetic analysis of the NOS1 gene in IHPS patients found 19 polymorphisms in the NOS1 coding region but found no statistically significant correlation with IHPS (Serra et al., 2011). SNP at −84 G/A on the promoter of NOS1 exon 1c (rs41279104) has been correlated with an increased risk for the development of IHPS, with a 30% decrease in NOS1 expression in 16 IHPS patients (Saur et al., 2004), although no such correlation was found in 54 familial and 28 sporadic cases of IHPS (Lagerstedt-Robinson, Svenningsson & Nordenskjöld, 2009), suggesting that the expression of NOS1 in IHPS is regulated by different factors other than SNPs. The −84 G/A polymorphism of NOS1 with IHPS was correlated in Caucasian subjects but was not found in the Chinese population, suggesting that genetic heterogeneity in different populations also plays a crucial role (Miao et al., 2010). Differences across population subjects in allelic frequency and linkage disequilibrium also allow for contrasting results. Analyses of DNAs obtained from three Swedish families with multiple affected members did not correlate with NOS1 locus (Söderhäll & Nordenskjöld, 1998); however, a study of 37 Swedish *vs* 31 British families with IHPS suggested positive correlation (Svenningsson et al., 2012). The major limitation of all studies was the limited number of subjects used. Study on larger samples with individuals from different races might help rule out the possibility of genetic heterogeneity due to different populations and direct us in analyzing the other factors responsible for NOS1 expression in IHPS. As discussed above, the mechanisms by which SNPs regulate gene expression constitute a large gap. To address this crucial gap, new technologies are required to fully elucidate the exact roles of SNPs in normal and disease settings.

## Conclusion and perspectives

**Figure 3:**
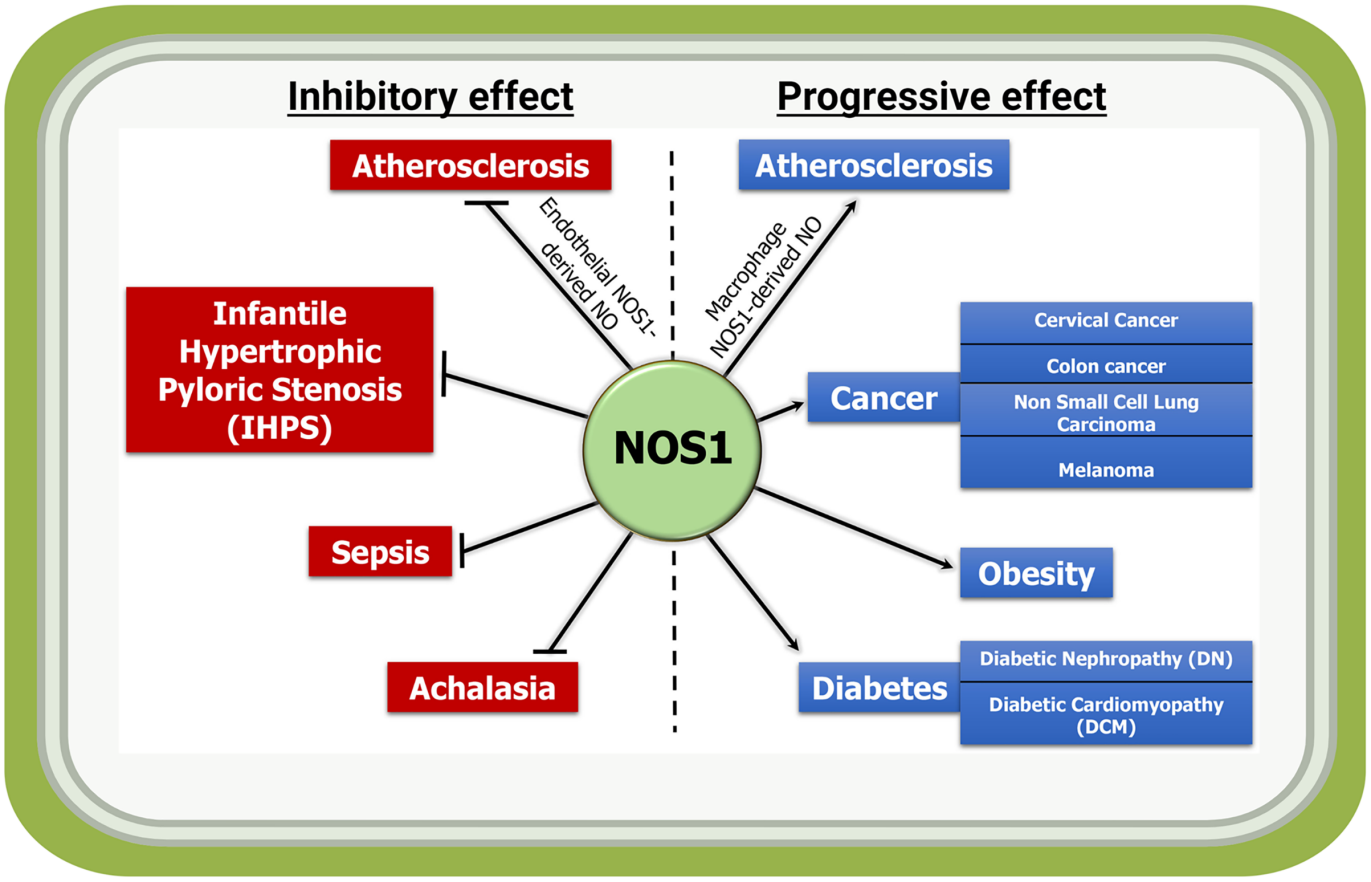
Inflammatory and anti-inflammatory activities of NOS1-derived NO in indicated diseases. NOS1-mediated production of NO acts as an inflammatory molecule and mediates the progression of disease such as macrophage NOS1-driven atherosclerosis, and in a subset of cancer, obesity and diabetes; while it acts as a protector in conditions such as endothelial NOS1-driven atherosclerosis, infantile hypertrophic pyloric stenosis (IHPS), sepsis and achalasia.

At the basal state, the NOS1 is the sole producer of NO in the brain, and involved in learning and memory development, and modulating synaptic plasticity. As NOS1 can be detected in non-neuronal tissues and organs including in cardiovascular disease, cancer, diabetes, and obesity (Fig. 3); therefore, we attempted to provide an overview of the roles of NOS1 in several pathologies to understand the importance of this molecule in different tissue and organ systems. The biological roles of NO in higher organism relies on its basal-state bioavailability and its temporo-spatial concentration. The organ- and cell type-specific NO production can also add to the complexity in signaling activities. Additionally, organelle specific localization or mis-localization could produce signaling cross-talks and unexpected phenotypes. However, the real workings of NOS1 mediated NO-modified proteins and how they transmit and modulate signaling mechanisms remain unknown.

Although found in a subset of tumors, NOS1 is not an oncogene or a tumor suppressor gene; in other words, NOS1 inhibition or activation in tumor cells may not inhibit the tumor cell proliferation. Conversely, whether the overexpression of NOS1 could transform NIH-3T3 fibroblast cells or the knockdown of NOS1 in tumor cells induce apoptosis, remain unknown. Thus, the precise role of NOS1 in tumor progression is not completely understood. Nevertheless, altered expression or misexpression of NOS1 and NO production thereby signaling cross-talks are likely to define pathophysiological states. Cell-specific *NOS1* gene knockouts studies are expected to clarify its exact function. Additionally, the use of small molecule agonist or antagonist for NOS1 and NO in combination with other tools that mediate downstream effect on *e.g*., NF-ƙB constitute a potential application for NOS1 mediated interventions in the complications associated with NO produced by NOS1.

Thus, the expression of NOS1 outside the brain in regulating many pathological states can no longer be neglected (Table S1). Therefore, sophisticated experiments delineating the signaling pathways and molecules regulated by NOS1-derived NO in non-neuronal tissues are needed. As the personalized medicine becomes more clinically relevant in combating certain ailments, elucidation of the complexity of NO signaling pathway will remain a topic of basic research. We feel that continued research on NOS1 in various pathophysiological situations will synthesize new knowledge as well as to identify therapeutic targets. State of the art techniques including bioelectronics to generate NO *in vivo*, assay for transposase-accessible chromatin with sequencing (ATAC-Seq), single-cell RNA-seq and biochemical methods combined with computational approaches will be required to address this problem more effectively.

## Supplemental Information

10.7717/peerj.13651/supp-1 Supplemental Information 1 Expression of NOS1 in non-neuronal pathophysiologies.

## Additional Information and Declarations

### Data Availability

The following information was supplied regarding data availability: This is a literature review.
