# Supplementary material for: The expanding roles of neuronal nitric oxide synthase (NOS1)
Source: PeerJ. 2022 Jul 7;10:e13651. doi: 10.7717/peerj.13651 (PMC9271274; doi:10.7717/peerj.13651)
Supplement: Supplemental Information 1 [file peerj-10-13651-s001.docx]

|  | **Diseases** | | **NOS1 derived NO function** | **NOS1 Source** |
| --- | --- | --- | --- | --- |
|  | **Cardiovascular** | **Atherosclerosis** | -NOS1 protects atherosclerosis by reducing regional area and its deletion in ApoE^-/-^ mice accelerates plaque formation. (Kuhlencordt et al., 2006; Schödel et al., 2009; Tsutsui, 2004)  -Disruption of NOS1 leads to enhanced inflammatory response by upregulating VCAM-1, proinflammatory cytokine expression and increased leukocyte recruitment. [(Chakrabarti et al., 2012)](https://www.zotero.org/google-docs/?zoHdd3)  -In macrophages, NOS1 derived NO mediates ox-LDL uptake, increases pro-inflammatory cytokines and CD40L expression, and induces CD40 expression on endothelial cells. [(Roy et al., 2020, 2021)](https://www.zotero.org/google-docs/?UIpoMz)  -NOS1 produces H**_2_**O_2_ & its production impairment leads to endothelial dysfunction in hypertension & atherosclerosis. [(Capettini et al., 2008, 2011; Silva et al., 2016)](https://www.zotero.org/google-docs/?Czm78A)  -Excessive ox-LDL uptake leads to uncoupling and downregulation of NOS1 in endothelial cells and leads to endothelial dysfunction by generation of superoxide and increases plaque formation. [(Navia-Pelaez et al., 2017)](https://www.zotero.org/google-docs/?86jl6g) | Macrophages and Endothelial Cells |
|  |  | **Cardiac**  **Hypertrophy** | -Deficiency of NOS1 leads to cardiac hypertrophy by increasing myocyte hypertrophy and ventricular stiffness and leads to increased mortality. [(Barouch et al., 2003; Cappola et al., 2003)](https://www.zotero.org/google-docs/?iBOac2)  -Basal NOS1 is required for normal cardiac functioning and S-nitrosylation of Ca**^2+^** regulating proteins as blockade of NOS1 decreases inotropic response and nitrosylation of LTCC, SERCA and RyR2 (Gonzalez et al., 2007; Irie Tomoya et al., 2015; Lima Brian et al., 2010; Treuer & Gonzalez, 2014; Vielma et al., 2016)  -In atherosclerotic children, 1% increase in NOS1 DNA methylation increases CIMT by 1.2μm [(Breton et al., 2014)](https://www.zotero.org/google-docs/?8fUCJn)  -NOS1 SNP rs3782218 leads to coronary heart disease and hypertension and SNP in *NOS1AP* is also associated with QT prolongation (delayed ventricular repolarization) and increase of sudden death in Type 1 QT syndrome patients  [(Levinsson et al., 2014,](https://www.zotero.org/google-docs/?EYdHXw) [Kao et al., 2009; Tomás et al., 2010)](https://www.zotero.org/google-docs/?w21jiN) | Cardiomyocytes |
| **2.** | **Cancer** | **Cervical** | -NOS1 regulates ABCG2 expression and its inhibition decreases ABCG2 & increases apoptosis. (Ding et al., 2019) | Cancer cells |
|  |  | **Pancreatic** | -NOS1 induce metastasis and stem-like property of pancreatic cancer cell line PANC-1 (Fujita et al., 2019) |  |
|  |  | **Melanoma** | -Amplification and expression of NOS1 locus in melanoma cells decrease the IFN signaling in PBMC by S-nitrosylation of HDAC2 at C262/C274 which decreases binding of HDAC2 to STAT1, reduces HDAC2 recruitment to ISGs promoter and decreases deacetylation of H4K16, thus mediating immune escape of tumor cells. [(Liu et al., 2014)](https://www.zotero.org/google-docs/?j1vOaW)  -NOS1 expression in metastatic melanoma cells negatively correlates with adoptive T-cell therapy. [(Liu et al., 2014)](https://www.zotero.org/google-docs/?MY5xrH) |  |
|  |  | **NSCLC** | -Expression of CXCL-14 and NOS1 is positively correlated with growth and invasion of NSCLC. (Ji et al., 2018)  -Oxidative stress leads to NOS1 induction & activation of NRF2 & HIF-1α signaling in CXCL-14 expressing CAF and inhibition of NOS1 inhibits the formation of tumors. ([Augsten et al., 2014)](https://www.zotero.org/google-docs/?IhRnG6) |  |
|  |  | **Colon** | -Mitochondrial NOS1 (mNOS1) increases SIRT3 activity which attenuates mitochondrial superoxide generation, decreases cisplatin-induced apoptosis and increases resistance to radiation and chemotherapeutic drugs. (Lai et al., 2016; Liu et al., 2015; Wang et al., 2019) |  |
| **3.** | **Diabetes** | **Diabetes**  **Nephropathy** | -Acute hyperglycemia induced hyperfiltration increases luminal glucose at Macula densa (MD) cells & leads to increase in NOS1 expression via SGLT1 which blunts tubulo-glomerular feedback response & promotes glomerular hyperfiltration. [(Zhang et al., 2019)](https://www.zotero.org/google-docs/?RMZDqD)  -Inhibition of NOS1 by SMTC in control and Streptozotocin-induced diabetic rats increased blood pressure and compared to control, diabetic rats showed enhanced renal hemodynamic response. [(Komers, Lindsley, et al., 2000](https://www.zotero.org/google-docs/?9mhrmy))  -STZ-induced diabetic rats have increased in the number of NOS1 positive cells & its inhibition by SMTC administration normalized the elevated GFR in diabetic rats. ([Komers, Oyama, et al., 200](https://www.zotero.org/google-docs/?EisKZ8)0, Komers et al., 2004)  -In nephroprotective role, long term NOS1 inhibition by SMTC delayed renal injury (proteinuria and glomerular sclerosis) in diabetic mice while no beneficial effect in non-diabetic rats. [(Komers et al., 2004)](https://www.zotero.org/google-docs/?KlsKDw) | Macula densa (MD) in Kidney |
|  |  | **Diabetes**  **Cardiomyopathy** | -BH4 prevents and reverse left-ventricular (LV) diastolic and systolic dysfunction associated with diabetes as the NOS1-derived NO mediates an increase in insulin-independent myocardial glucose uptake and utilization. [(Carnicer et al., 2021)](https://www.zotero.org/google-docs/?zdqbuz)  -NOS1 translocation from Sarcoplasmic reticulum (SR) to Sarcolemma caveolae decreases the nitrosylation of RyR2 and activates it to release unregulated Ca^2+^ generating arrhythmias. [(Gonzalez et al., 2007)](https://www.zotero.org/google-docs/?oB4xbl)  -NOS1-derived NO mediates β3-adrenoceptor (β3-AR) involved in altered positive inotropic response to β-adrenoceptor stimulation & play role in prognosis of this disease by β3-AR-NOS1-RyR2 pathway & Inhibition of NOS1 leads to normalization of adverse conditions. [(Amour et al., 2007; Birenbaum et al., 2008; Moens et al., 2010; Niu et al., 2012)](https://www.zotero.org/google-docs/?k6RzBT) | Cardiomyocytes |
| **4.** | **Obesity** |  | -Mice fed with high-fat diet have increased NOS1 expression in aorta due to leptin stimulation & its deficiency decreased NOS1 expression with increase in oxidoreductase (XOR) activity, an imbalance in nitroso-redox & generates myocardium dysfunction, cardiac hypertrophy, increased apoptosis of cardiac cells and decreased survival in obesity. Leptin treatment restores NOS1 activity. [(Sansbury& Hill, 2014; Saraiva et al., 2007)](https://www.zotero.org/google-docs/?Kq5Qzp)  -In pancreatic beta-cells, increased sensitivity and utilization to glucose is found in insulin-resistant rats and islets of obese humans which leads to insulin hypersecretion and increases production and dimerization of NOS1, but decrease its catalytic activity. [(Mezghenna et al., 2011)](https://www.zotero.org/google-docs/?2ka3BR)  -NOS1 SNPs rs2293048 and rs9658490 along with TCG and TGG haplotypes are associated with susceptibility to obesity. [(Park et al., 2016)](https://www.zotero.org/google-docs/?lR40Yt) | Aorta &  Pancreatic beta-cells |
| **5.** | **Sepsis** | **Peritonitis** | -NOS1 KO mice lead to increased migration of leukocytes but genetic deficiency of NOS1 leads to increased mortality. [(Cui et al., 2007)](https://www.zotero.org/google-docs/?B8qDVs) | Vascular tissue |
|  |  | **Experimental Sepsis** | -Blockade of NOS1 inhibits cGMP production in vascular tissue, decreases hypo-responsiveness and increases vasoconstriction in the sepsis model. [(Nardi et al., 2014)](https://www.zotero.org/google-docs/?Fe4yM5) |  |
| **6.** | **Achalasia** |  | -nNOS29 C/T polymorphism have positive correlation with progression of Achalasia by influencing the stability and expression of NOS1 mRNA. [(Singh et al., 2015)](https://www.zotero.org/google-docs/?izOeWM)  -Homozygosity of premature stop codon of NOS1 gene in 2 siblings with infant-onset achalasia resulted in defect in folding, binding of co-factor and NO production from NOS1. [(Shteyer et al., 2015)](https://www.zotero.org/google-docs/?viWF4r) | Smooth muscle cells of esophagus |
| **7.** | **Infantile Hypertrophic Pyloric Stenosis (IHPS)** |  | -NOS1 gene mutation between the 21st and 22nd exome in IHPS patient affects the splicing and decreases the expression of NOS1 mRNA. [(Jabłoński et al., 2016)](https://www.zotero.org/google-docs/?zJZBd5)  -84G/A SNP on promoter of NOS1 exon 1c (rs41279101) have positive correlation with IHPS progression as 30% decrease in NOS1 expression was found in 16 IHPS patients, but results were not able to translate on larger subjects. [(Lagerstedt-Robinson et al., 2009; Saur et al., 2004)](https://www.zotero.org/google-docs/?GlAzQ6)  -84G/A SNP polymorphism of NOS1 with IHPS progression was positively correlated in Caucasian subjects but not on Chinese subjects, suggesting genetic heterogeneity. [(Miao et al., 2010)](https://www.zotero.org/google-docs/?O2s5zx)  -DNA of 37 Swedish and 31 British families suffering with IHPS studied for linkage disequilibrium of NOS1 locus found positive correlation with IHPS progression, but was not correlated in 3 Swedish families in different studies. [(Söderhäll&Nordenskjöld, 1998; Svenningsson et al., 2012)](https://www.zotero.org/google-docs/?3CxCbx)  -19 polymorphisms of NOS1 coding region in IHPS patients showed no correlation with IHPS prognosis. [(Serra et al., 2011)](https://www.zotero.org/google-docs/?c0gpV7) | Pyloric muscle cells |
